# Accelerating Generation and Impacts of Research Evidence to Improve Women Veterans’ Health and Health Care

**DOI:** 10.1007/s11606-022-07607-0

**Published:** 2022-08-30

**Authors:** Elizabeth M. Yano, Naomi Tomoyasu

**Affiliations:** 1 VA HSR&D Center for the Study of Healthcare Innovation, Implementation and Policy, VA Greater Los Angeles Healthcare System, Los Angeles, CA USA; 2 Department of Health Policy and Management, University of California, Los Angeles (UCLA) Fielding School of Public Health, Los Angeles, CA USA; 3 Department of Medicine, UCLA Geffen School of Medicine, Los Angeles, CA USA; 4 VA Health Services Research and Development (HSR&D) Service, Office of Research and Development, Veterans Health Administration, Washington, D.C. USA

In 1990, the National Institutes of Health established the Office of Research on Women’s Health in recognition of “an astonishing lack of knowledge on conditions that are unique to or more prevalent” among women.^1^ Within a decade, as women Veterans became the fastest-growing group of new users, the Veterans Health Administration (VA) was faced with the challenge of accelerating delivery of comprehensive women’s health care in a system historically designed to serve men’s health-care needs. This shift was made more difficult given the dearth of knowledge about women Veterans’ health and health-care needs and their numerical minority (< 8% of all VA users), complicating their inclusion in VA research in sufficient numbers to explore subgroup differences.^2^

As the largest national integrated health-care delivery system in the U.S.A., the VA has long had an intramural research program spanning biomedical, clinical, rehabilitation, and health services research. By the early 2000s, VA added a focus on implementation science, hastening translation of research into evidence-based practice and policy. As demand for evidence to support improved care delivery grew, VA developed its first enterprise-wide women’s health research agenda (2004), later focusing on women’s health services research and development (HSR&D) (2011).^3,4^ VA also funded the VA Women’s Health Research Network (WHRN) in 2010 to build women’s health research capacity through structured support for a national consortium of researchers (e.g., support for training, mentorship, collaborative research development, dissemination) and a practice-based research network (PBRN) to foster inclusion of women in VA research.^5^

With a national research agenda and WHRN support, we observed a substantial increase in the volume of women’s health-centric proposal submissions and increased inclusion of women in observational and interventional studies, enabling appraisal of gender differences. Early establishment of a scientific review board that focused on women’s health contributed to early growth in funding, which continued after that board was expanded to include other topics (e.g., access), having created a critical mass of women’s health expertise among reviewers. The result was a dramatic increase in funding of women Veterans’ research (over $40 million in the last decade). Since women Veterans’ health remains a high priority for VA and key knowledge gaps remain, continued efforts to ensure sufficient numbers of high-quality proposals are submitted and funded will be an important objective for VA HSR&D.

In parallel, the women’s health research agenda’s focus on topics across the lifespan, and WHRN’s support of national collaborative research work groups anchored in the agenda, led to a substantial diversity of research topics taken on by the research community.^4^ We have observed expansion of research in the following areas:

- *Mental health*. Research has been funded on women Veterans’ trauma, intimate partner violence, and suicide prevention, as well as their risks and resiliency. For example, as research illuminated that women Veterans’ suicide rates were nearly double that of non-Veteran civilian women, WHRN developed an ad hoc work group that through collaborative research development (conference, strategic planning work group calls, methods consults) yielded eight studies in just over 2 years.
- *Reproductive health*. The volume of VA reproductive health research has grown dramatically, including new studies of fertility, pre-conception care, maternity care quality, birth outcomes, uterine fibroids, and menopause, among other topics.
- *Rural health/access*. As over one in four women Veterans live in rural and highly rural areas, research on their health-care needs has grown, including work on telehealth options to improve access, given that longer drive times are associated with greater attrition from VA care.
- *Post-deployment health*. While VA’s investment in post-deployment health research, supporting transitions from military to civilian life, has always been substantial, new research was funded on interventions to facilitate post-war access of Reserve and National Guard servicewomen and enhance their mental health treatment engagement.
- *Primary care/prevention*. Prior to HSR&D’s investment in WHRN, only one study had focused on women’s preventive care (specifically, mammography screening). With the launch of a primary care/prevention work group, several studies examined gender differences in cardiovascular disease (CVD) risks and gender-tailored CVD risk reduction, given gender differences in presentation, treatment preferences, and outcomes. Concurrently, VA rolled out the patient-centered medical home model as the new primary care delivery model nationwide, with a women’s health–focused version in national policy. VA researchers studied how women Veterans’ primary care and women’s health services were organized and how variations influenced care quality and patient experience. These research findings informed a subsequent trial of an evidence-based quality improvement (EBQI) approach to gender-tailoring primary care to meet women Veterans’ needs; EBQI was subsequently adopted by the VA Office of Women’s Health as a national strategy for improving comprehensive women’s health care.
- *Complex chronic conditions*. Women Veterans who use the VA also often have complex care needs, given physical and mental health comorbidities that complicate routine care management, and may require supportive care coordination across specialists within and outside the VA. VA research has responded with new studies of gender differences in chronic pain, sleep disorders (e.g., insomnia), and trauma-informed care models, as well as studies of virtual care delivery.

With VA HSR&D funding, WHRN also focused on research dissemination. Early on, VA women’s health researchers described barriers to publication: journals that routinely published research on Veterans were less familiar with research focused on *women* Veterans, while those focused on women’s health traditionally lacked reviewers with *Veteran* expertise. HSR&D responded by funding journal supplements like this one to ensure that this work has the visibility needed to build traction and hallmark the science needed to inform evidence-based changes in practice and policy. Aided by these supplements, alongside increased women’s health research funding, came a concomitant gain in publications: more women Veterans’ research papers were published in the 5 years since the agenda-setting effort than the entire 25 previous years combined, followed by an over 125% increase in the next 7 years.^6,7^ The supplements also had the effect of demonstrating VA’s commitment to women’s health research, further encouraging VA researchers to conduct women Veterans’ research, examine gender differences, and consider gender-tailoring of interventions and implementation. VA HSR&D also funded field-based research conferences focused on women Veterans’ research, bringing partners from the U.S. Departments of Defense, Health & Human Services, and Labor, among other federal partners, together with VA leaders and researchers to infuse practice and policy debates with scientific evidence. We supported Spotlight on Women’s Health national webinars highlighting research results and priority needs for research evidence, often integrating system partners as discussants to anchor research findings in the context of VA health care delivery. Over time, demand for real-time briefings for VA system leaders and external stakeholders (e.g., congressional briefings, women Veteran advocacy groups) increased. WHRN also generated brief lay language research summaries on priority topics (e.g., syntheses of research on women Veterans’ suicide risks, harassment, health-care needs in rural areas).

In parallel with building capacity through the national consortium of researchers, WHRN also built capacity to facilitate recruitment of women Veterans in VA research and implementation of research into practice through the PBRN.^5^ Having grown from four to 32 to now over 75 VA medical centers as members, the PBRN has supported the conduct of nearly 90 studies. Lessons learned from this work have influenced enrollment in VA clinical trials as well, leading to funding of a national hub-and-spoke model for increasing women’s inclusion in and their equitable benefit from VA’s research investments.

To accelerate research impacts, VA HSR&D also funded the Women Veterans’ Healthcare CREATE Initiative (2013-18), a five-study collaborative research program partnered with the VA Office of Women’s Health and other system leaders to accelerate implementation of comprehensive women’s health care.^8^ The VA Quality Enhancement Research Initiative (QUERI) also increased funding of rigorous partnered evaluations and implementation trials of gender-tailored evidence-based practices. For example, the EMPOWER QUERI (2015-20) engaged operational partners and women Veterans in tailoring and implementation of new models of diabetes prevention, CVD risk reduction, and primary care-mental health integration.^9^ The focus on bringing research from descriptive and observational studies to interventions and implementation was also leveraged by WHRN’s addition of a multilevel stakeholder engagement arm, which included researchers, frontline providers, system leaders, and, importantly, women Veterans themselves. Research impacts have been enabled in large part because of this partnered nature of VA research and the embedded nature of our research workforce (e.g., the majority of VA researchers are clinicians who also deliver care in the system), contributing to VA’s evolution as a learning health system.

VA HSR&D’s embrace of collaborative research development in partnership with system leaders and in service of improving Veterans’ care has paid substantial dividends. In addition to demonstrated impacts on women’s health practice and policy, we have applied the WHRN model for building national consortia to top priority research needs (e.g., suicide prevention, chronic pain/opioids, virtual care), focusing resources, building synergies, and hopefully having comparable research impacts. At the same time, we are mindful that there is much work ahead. While the NIH Office of Research on Women’s Health has long required inclusion of women in research, it remains challenging to get researchers to publish gender differences that could be used to tailor future interventions to meet women’s distinct needs outside let alone inside VA. While VA women’s health research funding has grown, we are still only tackling a fraction of the work needed. A recent RAND study demonstrated that increasing the investment in women’s health research would have an extraordinary return on investment (ROI). For example, if the NIH budget for research on coronary artery disease in women was doubled from its current $20 million, we could expect a return on investment of *9500%*.^10^ While the VA version of this kind of ROI analysis is unknown, we have already seen the positive impacts of the past decade’s focused attention on women Veterans’ research, with the current journal supplement speaking volumes to the strides made. We need to actively identify ongoing knowledge gaps and underfunded areas of inquiry, while simultaneously pushing toward intervention and implementation. We look forward to the future as we continue to break new ground in accelerating the generation and impacts of research evidence to systematically improve women Veterans’ health and health care.

## Authors contributors

The authors would like to thank Susan M. Frayne, MD, MPH, Center for Innovation to Implementation, VA Palo Alto Healthcare System, and Alison B. Hamilton, PhD, MPH, Center for the Study of Healthcare Innovation, Implementation and Policy, VA Greater Los Angeles Healthcare System, for their joint leadership of the VA Women’s Health Research Network (WHRN) and contributions to the field.
